# Audit on prolactin monitoring for patients on oral risperidone, intramuscular risperidone, and intramuscular paliperidone

**DOI:** 10.1192/bjo.2021.216

**Published:** 2021-06-18

**Authors:** Mohamed Bader

**Affiliations:** Aneurin Bevan University Healthboard

## Abstract

**Aims:**

The aim of this audit was to investigate whether sufficient Prolactin monitoring was completed in a patient sample in the Torfaen area of Aneurin Bevan University Health Board. This audit targetted patients an oral or intra-muscular formulation of Risperidone in the year 2018 with the hypothesis that Prolactin monitoring is done less frequently than recommended.

**Background:**

Risperidone is the anti-psychotic drug most frequently associated with hyperprolactinemia which is often asymptomatic but can present with symptoms of oligomenorrhea, amenorrhea, galactorrhea, decreased libido, infertility, and decreased bone mass in women. Men with hyperprolactinemia may present with erectile dysfunction, decreased libido, infertility, gynecomastia, decreased bone mass, and rarely galactorrhea. The BNF advises monitoring of Prolactin at baseline, after 6 months, and then annually.

**Method:**

Retrospective review of 150 patients’ clinical letters to identify if they are on the above medications, using the local digital records system EPEX. Emails were also sent to community psychiatric nurses asking them if they could highlight any patients they were caseholding on the above medication. Depot clinic lists were also examined. Patients identified as being on the above medication had their blood tests reviewed on the online system Clinical Workstation (CWS) to determine whether they had their Prolactin level tested. A single spot sample of all patients on Talygarn ward in January 2019 was also included.

**Result:**

1. 28 Risperidone

2. 23 of 28 never had any Prolactin measurements

3. 2 of 28 patients had the appropriate level of monitoring done for the year of 2018

a. One patient complained of Galacotorrhea

b. Another patient had baseline done while on the ward and isn't due for any further monitoring at the time of writing.

**Conclusion:**

The above results identify that Prolactin monitoring is not being routinely completed for patients on the studied medication at an acceptable compliance level. Limitations around utitlity of prolactin monitoring may be the contributing factors; eg. Prolactin levels or medication dose may not be positively associated with adverse effects.. Further efforts were made to highlight the importance of baseline prolactin monitoring, as well as including a baseline Prolactin as an admission blood test for patients presenting with psychotic symptoms or on an anti-psychotic. A complete audit of metabolic monitoring and Prolactin levels for all patients on anti-psychotics would be an appropriate next step.

